# Early-onset psychosis as a manifestation of Fahr’s syndrome: a case report

**DOI:** 10.1192/j.eurpsy.2025.848

**Published:** 2025-08-26

**Authors:** M. Uzar, W. Zwolińska, A. Słopień

**Affiliations:** 1 Department of Child and Adolescent Psychiatry, Poznan University of Medical Sciences, Poznan, Poland

## Abstract

**Introduction:**

Fahr’s syndrome/disease is a rare neurological disorder characterized by calcification in brain areas such as the basal ganglia, cerebellum, and subcortical white matter. The disorder may manifest with both neurological and psychiatric symptoms. Fahr’s syndrome has a genetic basis and is inherited in an autosomal dominant manner. It may also develop as a result of injuries, carbon monoxide poisoning, or hormonal disturbances. If the calcification is idiopathic, the term Fahr’s disease is used.

The neurological symptoms of the condition include motor disorders, delirium, and dementia. However, it may also manifest in psychiatric symptoms such as anxiety, obsessive-compulsive symptoms, cognitive decline, psychotic symptoms, and mood disorders. Available data indicate that the early onset of Fahr’s syndrome symptoms is associated with a worse prognosis compared to the later onset.

**Objectives:**

Our work aims to emphasize the necessity to look for/exclude the neurological basis of early forms of psychosis.

**Methods:**

Herein, we present a case report of a thirteen-year-old patient hospitalized in the Department of Child and Adolescent Psychiatry in Poznań because of persistent psychotic symptoms. Due to severe headaches, episodes of vomiting, balance disorders, and hallucinations experienced before psychiatric hospitalization, the patient was referred to a neurological ward where imaging tests were performed revealing calcifications in both globi pallidi. Consequently, Fahr’s syndrome was diagnosed.

During the patient’s hospitalization in the Department of Child and Adolescent Psychiatry, her mother reported that the patient had exhibited aggressive and self-aggressive behavior. Moreover, the patient’s school performance had been deteriorating for approximately two years before being admitted to the hospital. The girl described visual hallucinations (seeing figures) and auditory hallucinations (thought echo and hearing voices telling her to harm herself) that had been occurring for over a year. She expressed delusions of reference and delusions of thought insertion.

Interestingly, during the further diagnostic process, similar neurological changes as in the patient were found in her mother’s brain. This was unexpected because it was the patient’s father who was treated for schizophrenia (for formal reasons, it was not possible to check whether he also had neurological changes).

**Results:**

The patient was diagnosed with both schizophrenia and Fahr’s syndrome. Pharmacotherapy was implemented (olanzapine and then additional aripiprazole), and she was also provided with psychotherapeutic care. The implemented treatment has led to only partial improvement.

**Conclusions:**

Further studies on Fahr’s syndrome are needed to better understand the disease course across the lifespan. Neuroimaging seems essential in early-life psychosis to exclude possible neurological basis of the condition.

**Disclosure of Interest:**

None Declared

